# Work disability patterns before and after incident acute myocardial infarction and subsequent risk of common mental disorders: A Swedish cohort study

**DOI:** 10.1038/s41598-019-52487-w

**Published:** 2019-11-06

**Authors:** K. Bokenberger, S. Rahman, M. Wang, M. Vaez, T. E. Dorner, M. Helgesson, T. Ivert, E. Mittendorfer-Rutz

**Affiliations:** 1 Division of Insurance Medicine, Department of Clinical Neuroscience, Karolinska Institutet, Stockholm, Sweden; 2 Department of Social and Preventive Medicine, Centre for Public Health, Medical University of Vienna, Vienna, Austria; 3 Department of Molecular Medicine and Surgery, Karolinska Institutet and Heart and Vascular Theme, Karolinska University Hospital, Stockholm, Sweden

**Keywords:** Myocardial infarction, Depression, Epidemiology, Risk factors

## Abstract

This study investigated the extent to which work disability patterns including sickness absence and disability pension (SA/DP) before and after acute myocardial infarction (AMI) were associated with subsequent common mental disorders (CMDs) such as depression and anxiety in AMI patients without previous CMD. Total 11,493 patients 26–64 years with incident AMI during 2008–10 were followed up for CMD (measured as antidepressant prescription) through 2013. Four SA/DP trajectory groups during the 3-years pre-AMI and 1-year post-AMI were identified. Hazard ratios (HRs) with 95% confidence intervals for subsequent CMD were estimated in Cox models. Higher pre-AMI SA/DP annual levels (>1–12 months/year) were associated with 40–60% increased CMD rate than the majority (78%) with low increasing levels (increasing up to 1 month/year). Regarding post-AMI findings, constant high (~25–30 days/month) SA/DP levels within the first 3 months was associated with a 76% higher CMD rate, compared to constant low (0 days/month). A gradually decreasing post-AMI SA/DP pattern over a 12-month period suggested protective influences for CMD (HR = 0.80). This is the first study to demonstrate that pre- and post-AMI work disability patterns are associated with subsequent CMD risk in AMI patients. Work disability patterns should be considered as an indicator of AMI prognosis in terms of CMD risk.

## Introduction

Acute myocardial infarction (AMI) is the leading cause of premature death worldwide^1^. In Sweden, approximately 26,600 people suffered from an AMI in 2015^2^, a third among whom were of working age^3^. Following an AMI, sickness absence (SA) is commonly prescribed as a rehabilitation measure for patients^4^. In Sweden, 4–8 weeks of SA are recommended for AMI without complications^4^. For AMI cases with complications, work ability may be permanently reduced, resulting in the patient being granted disability pension (DP) by the Social Insurance Agency^4^.

Common mental disorders (CMDs) such as depression, anxiety, and stress-related mental disorders, which are highly prevalent globally^5^, are associated with increased risk of AMI^6–10^ and mortality^11^. Over the last decade, CMDs have become the most common diagnoses for work disability, i.e. SA or being granted DP, in working populations^12,13^. In addition to the substantial economic costs incurred by work disability payouts, such labour market marginalization is tied to adverse health behaviour and social isolation^14^, factors which in turn heighten vulnerability for CMDs^15^. Further, the onset of CMDs may be triggered after an AMI^16^, a life-threatening event that is perceived as stressful by those affected^17^. Bridging these topics, recent research has demonstrated that patients suffering ischemic heart disease and CMDs have higher DP rates compared to those separately affected by ischemic heart disease or CMDs^18^.

AMI patients sometimes have sickness absence preceding the cardiac event due to cardiovascular causes or other diseases^19,20^ and particularly after the event due to rehabilitation purposes^20,21^. In the Swedish health care system, AMI patients are generally followed up on cardiac rehabilitation outcomes, including return to work status, at approximately 2 months and again at 1 year after the event by the attending physician^22^. Earlier studies have demonstrated heterogeneity in work disability trajectory patterns, with sociodemographic factors such as female sex and low education, as well as comorbid musculoskeletal and mental disorders driving the variation in these patterns^20,23^. However, there is a lack of research on the impact of pre- and post-AMI patterns of sickness absence and disability pension (SA/DP), which may be a useful marker in terms of CMD prognosis.

## Aim

The present study aims to examine the extent to which patterns of work disability, in terms of SA/DP, before and after AMI are associated with subsequent CMDs in AMI patients without prior CMDs taking into consideration sociodemographic and medical factors.

## Methods

### Study design and population

This prospective population-based nationwide study comprises a cohort of 16,983 patients aged 26–64 years who had a first AMI at baseline (T0) during 2008–2010. AMI patients were identified as those who received inpatient care with a main diagnosis (International Classification of Diseases version 10 (ICD-10) code of I21) according to the National Patient Register. Those who had an earlier AMI prior T0 sometime between 1987 and 2008 (n = 1,914) and those who died within 30 days after the AMI (n = 424) were excluded. After further exclusion of subjects with CMD prior T0 (n = 3,152), the main cohort comprised 11,493 AMI patients without previous CMD. To examine post-AMI work disability patterns in relation to CMD risk, a subsample of 10,642 patients still alive and who had not developed CMD within 1 year after AMI were followed up starting from 1 year after AMI (T1). Previous CMD was defined as those with prescribed and dispensed antidepressants or had inpatient or specialised outpatient care for depression, anxiety, or stress-related disorders prior T0 or T1, respectively. Both study populations were followed up until date of post-AMI CMD (see *CMD measures*), death, emigration, or end of follow-up on December 31, 2013, whichever came first.

### Register data

Several national registers were linked to the study cohort with the assistance of the unique personal identification number assigned to all Swedish inhabitants. The following registers were included: (i) Longitudinal Integration Database for Health Insurance and Labour Market Studies (LISA) held by Statistics Sweden: includes data on age, sex, country of birth, educational level, type of living area, family situation, and year of emigration; (ii) MicroData for Analysis of the Social Insurance database (MiDAS) held by the Social Insurance Agency: includes data on length, extent and diagnosis of SA/DP. In Sweden, all residents from the age of 16 years with a minimum annual income from work or unemployment are eligible to receive SA benefits from the public Social Insurance Agency (SIA) in case of reduced work ability due to disease or injury. Those, 30–64 years of age with permanent work disability can be granted DP, and those 19–29 years of age with temporary work disability can be granted temporary DP^24^. Both SA and DP can be granted for partial (25%, 50%, 75%) or complete (100%) work disability; (iii) National Patient Register held by the National Board of Health and Welfare (NBHW)^25^: includes records of date and diagnosis of inpatient care since 1987 and specialised outpatient care since 2001; (iv) Prescribed Drug Register held by the NBHW: comprises records of date, type and dosage of the drug dispensed; and v) Cause of Death Register held by the NBHW: includes data on date of death.

### CMD measures

Measures for depression, anxiety, and stress-related disorders were derived based on ICD codes (F32-F33, F40-F42, and F43, respectively) from the National Patient Register. A measure for prescribed antidepressant medication was created based on Anatomical Therapeutic Chemical Classification System codes (N06A) from the Prescribed Drug Register. The main outcome measure for CMD (yes/no) after AMI was based on purchases of antidepressants. For sensitivity analyses, we also defined CMD as antidepressant prescription or specialised health care diagnosis of depression, anxiety, or stress-related disorders. In order to exclude patients with CMD prior to AMI, both diagnostic and prescription data were used for the measurement.

### Work disability

Pre-AMI work disability trajectories were based on combined net days of SA/DP per annum. Post-AMI work disability trajectories were based on combined net days of SA/DP per month. Net days for work disability were calculated by multiplying the length and extent of SA/DP days. For instance, if a person was on SA/DP for 50% for 2 days, then the length of SA/DP would be equal to SA/DP for 100% for 1 day, that is the individual would be considered as having ‘1’ net day of SA/DP.

Derivation of work disability trajectories are described in *Statistical methods*.

### Covariate measures

Sociodemographic characteristics comprised sex, age, educational level, family situation, area of living, and country of birth that were measured at the end of the year preceding AMI (see categorization in Table 1). Medical characteristics (Table 2) measured within the three years preceding the first AMI including somatic (co-)morbidities and other mental comorbidities were based on inpatient and specialised outpatient care (main and side diagnoses). Conditions included stroke (ICD-codes: I60, I61, I63, I64), hypertension (ICD-codes: I10), diabetes mellitus (ICD-codes: E10-E14), musculoskeletal disorders (ICD-codes: M00-M99), renal insufficiency (ICD-codes: N17-N19), cancer (ICD-codes: C00-D48), and other somatic disorders which included infectious diseases, blood disorders, diseases of the metabolic, nervous, ocular, auditory, respiratory, digestive, integumentary, and genitourinary system, conditions related to pregnancy, congenital disorders, injuries and poisonings (ICD-codes: A00-B99, D50-E07, E15-E90, G00-H95, J00-L99, N00-N16, N20-O75, O85-O99, Q00-T98). Mental disorders other than CMDs included bipolar disorder, schizophrenia, mood disorders not including depression, neurotic and somatoform disorders not including anxiety disorders, behavioural and emotional disorders, personality disorders, developmental disorders, and unspecified mental disorders (ICD-codes: F31, F20-29; F34-F39, and F44-F99). Measures of AMI-related characteristics (Table 2) included: type of infarction (classified as ST-elevation myocardial infarction (STEMI, ICD-codes: I21.0-I21.3), non-ST-elevation myocardial infarction (NSTEMI, ICD-code: I21.4) or unspecified (ICD-code: I21.9)) as an indicator of severity of AMI; and type of coronary revascularisation which was classified as percutaneous coronary intervention (PCI) (FNG00-FNG05), coronary artery bypass grafting (CABG) (FNA-FNF, FNG30, FNW96) and others (i.e. other intracardiac procedures or missing information) according to the Classification of Surgical Procedures.

**Table 1:** Sociodemographic characteristics of the main cohort of patients with a diagnosis of AMI from inpatient care during 2008–2010 in Sweden.

	N = 11,493	%
**Sociodemographic characteristics** ^a^		
***Age***		
26–45	1,043	9.0
46–55	3,540	30.8
56–64	6,910	60.1
***Sex***		
Men	9,142	79.5
Women	2,351	20.5
***Country of birth*** ^b^		
Sweden	9,354	81.4
Other Nordic countries	619	5.4
EU25 (excluding Nordic countries)	331	2.9
Other countries	1,189	10.3
***Education (years)***		
Compulsory (≤9)	3,367	29.3
High school (10–12)	5,627	49.0
University (>12)	2,499	21.7
***Type of living area*** ^c^		
Big cities	3,404	29.6
Medium sized cities	4,078	35.5
Small towns/villages	4,011	34.9
***Family situation*** ^d^		
Married living without children	3,236	28.2
Married living with children	3,950	34.4
Single living without children	3,782	32.9
Single living with children	525	4.6

Abbreviations: AMI = Acute myocardial infarction.

^a^Measured on December 31st of the year preceding acute myocardial infarction.

^b^Other Nordic countries include Denmark, Finland, Iceland, and Norway. Missing data is considered as other countries.

^c^Type of living area: big cities (Stockholm, Gothenburg and Malmö); medium-sized cities (cities with more than 90 000 inhabitants within the 30 km distance from the centre of the city); small cities/villages/rural.

^d^Married includes all living with partner or cohabitant. Single includes divorced, separated, or widowed. Missing data is considered single living without children.

**Table 2:** Medical and AMI-related factors of all patients with a diagnosis of AMI from inpatient care during 2008–2010 in Sweden.

	N = 11,493	%
***Type of infarction*** ^a^		
STEMI	5,165	44.9
Non-STEMI	4,053	35.3
Unspecified	2,275	19.8
***Coronary revascularisation characteristics at T0*** ^b^		
Percutaneous coronary intervention	8,159	71.0
Coronary artery bypass grafting	262	2.3
Others	3,072	26.7
***Somatic and mental disorders other than CMD 3 years prior and up to T0*** ^b^		
Musculoskeletal disorders	1,596	13.9
Diabetes mellitus	1,750	15.2
Renal insufficiency	148	1.3
Hypertension	3,881	33.8
Stroke	123	1.1
Cancer	654	5.7
Other somatic disorders	7,344	63.9
Other mental disorders	814	7.1
***CMD after AMI***		
CMD (Antidepressant prescription)	1,401	12.2

Abbreviations: AMI = Acute myocardial infarction. STEMI = ST-elevation myocardial infarction. CMD = Common mental disorder.

^a^ST-elevation myocardial infarction. Non-ST-elevation myocardial infarction.

^b^T0 refers to the date of the first AMI (baseline) during 2008–2010.

### Statistical methods

First, pre- and post-AMI work disability trajectories were identified using latent group-based trajectory modelling. Selection of the amount of trajectory groups was based on Bayesian information criterion fit estimates and the authors’ observations that including more than four groups in the trajectory models did not yield additional information or insight and likely led to overfitting of models. Group probabilities were calculated for each of the trajectory variables and yielded probabilities >90%, indicating that the trajectory variable fitted the data adequately well. Creation of the work disability trajectory measure has been described previously^20^. In brief, using this method, one may identify subgroups of individuals with distinct trajectories over time and estimate the proportion of persons in each group^26^. A work disability measure with four trajectory groups during the 3-year period preceding an AMI (“low increasing”, “middle increasing”, “variable”, “constant high”) and during the 1-year period following an AMI (“constant low”, “steeply decreasing”, “gradually decreasing”, “constant high”) were derived.

Second, crude and adjusted (separately and fully for sociodemographic and medical characteristics) hazard ratios (HRs) with 95% confidence intervals (CIs) were estimated for CMDs using Cox regression models. Main analyses entailed examining pre-AMI work disability trajectories in relation to subsequent CMDs, defined as purchases of antidepressant medication. Pre-AMI work disability trajectories were examined in the sample of patients with a first AMI in 2008–2010 (N = 11,493) and post-AMI work disability were examined in the sample of patients who survived 1 year after the first AMI and had not developed CMD (N = 10,642). Additionally, in sensitivity analyses, we examined models with a more broadly defined measure of CMD as an outcome measure as mentioned above.

### Ethical approval

The study has been approved by the Regional Ethical Committee of Stockholm and by the ethical committees of Statistics Sweden, the National Board of Health and Welfare, and the Social Insurance Agency. After legal review, the data are only made available for researchers in the group who meet the criteria for access to this type of sensitive data. These procedures follow the Swedish Ethical Review Act, the Personal Data Act, and the Administrative Procedure Act. Data are derived from different registers. Therefore, no personal contact with the individuals is established. The integrity of the persons is secured through de-identification of the individual information by the data providing agencies. Results are presented on group level without any possibility of backward identification, as in numerous previous publications derived from this database. The research group applies high standards of data safety.

### Informed consent

The study population was based on linkage of several public national registers. Ethical vetting is always required when using register data in Sweden. The ethical vetting is performed by regional ethical review boards and the risk appraisal associated with the Law on Public Disclosure and Secrecy is done by data owners (Statistics Sweden, the National Board of Health and Welfare, and the Social Insurance Agency). The ethical review boards can however waive the requirement to consult the data subjects (or in case of minors/children the next of kin, careers or guardians) directly to obtain their informed consent, and will often do so if the research is supported by the ethical review board and the data has already been collected in some other context. This means that for this specific study no written informed consent was required from participants (or next of kin/caregiver in the case of children) for their clinical records to be used. Patient records/information were anonymized and de-identified prior to analysis by the authority, Statistics Sweden, the National Board of Health and Welfare, and the Social Insurance Agency, who were responsible for data linkage. Researchers received de-identified data. This study was approved by the Regional Ethical Committee of Stockholm, and by the Ethical Committees of the data providers.

## Results

### Characteristics of AMI patients

Descriptive statistics are presented for the main cohort with no CMD prior T0 in Table 1a,b. The majority of AMI patients were 56–64 years old (median age of 57 years), men, born in Sweden, those with high school level education, and married and living with children.

**Figure 1:**
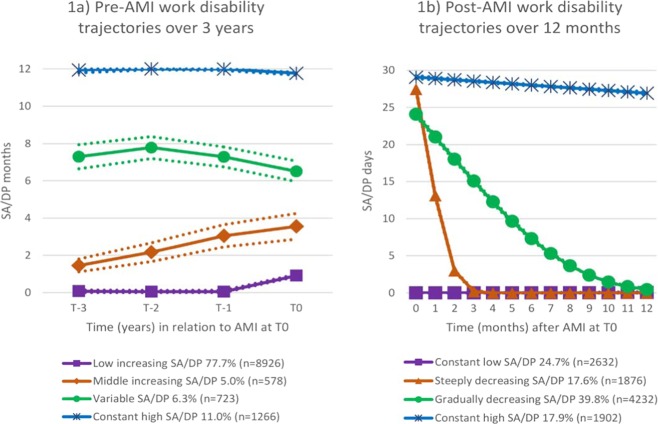
(**a**) Pre-acute myocardial infarction (AMI) trajectories of work disability in patients with a first AMI in 2008–2010 (N = 11,493); (**b**) Post-AMI trajectories of work disability in surviving patients within 1 year after the first AMI (N = 10,642). SA/DP = Sickness absence/disability pension.

Regarding work disability patterns, four different trajectories of pre-AMI work disability over a 3-year period and post-AMI work disability over a 12-month period were identified. The most common pre-AMI trajectory group, represented by 78% of patients, was characterized by “low increasing” annual levels wherein SA/DP levels increased from zero to 1 month (Fig. 1a). Eleven percent of patients made up the second most common pre-AMI trajectory group which was characterized by “constant high” (~12 months/year) SA/DP levels. “Variable” (vacillating between 6–8 months/year) and “middle increasing” (increasing from >1 month/year at T-3 to 4 months/year at T0) trajectory groups represented the rest of the cohort. Regarding post-AMI trajectory patterns, the majority of patients (40%) demonstrated “gradually decreasing” monthly levels of work disability wherein SA/DP levels started high at ~25 days/month and tapered off to 0 days/month by 12 months post-AMI (Fig. 1b). Those with “constant low” monthly levels (0 SA/DP days/month throughout the 12-month period) were represented by 25% of patients. Finally, groups with “constant high” (~25–30 days/month) and “steeply decreasing” SA/DP levels within the first 3 months each comprised about 18% of patients.

Median follow-up time was 4 years (max 6 years) for the cohort with no CMDs prior T0, and 3.3 years (max 5 years) for the cohort with no CMDs prior 1 year after AMI. Besides this, the distribution of sociodemographic and medical characteristics was practically the same between the two cohorts.

### Pre-AMI patterns of work disability and risk of CMD

“Middle increasing” levels of pre-AMI work disability over the course of 3 years were associated with a 60% higher rate of CMD while “variable” (i.e. increasing and decreasing levels) and “constant high” levels were associated with about a 40% increased CMD rate compared to individuals following “low increasing” levels of pre-AMI work disability (Table 3). Overall, pre-AMI findings point to moderately increasing levels of work disability over time being more strongly associated with greater CMD risk rather than constant high levels of work disability. Results were similar when models were adjusted separately and altogether for AMI-related factors and comorbidities, including somatic and mental disorders other than CMDs, such as bipolar disorder and schizophrenia. Overall, there was a modest attenuation in estimates for the association of work disability patterns with subsequent CMDs in fully adjusted models compared to that in crude models.

**Table 3:** Hazard ratios (HR) and 95% Confidence Intervals (CI) for common mental disorders (CMDs) defined as antidepressant prescription following a diagnosis of AMI from inpatient care in 2008–2010 in Sweden in persons without prior CMD^a^ (N = 11,493)

	CMD	Model 1^c^	Model 2^d^	Model 3^e^	Model 4^f^
	n (%)	HR (95% CI)			
***Trajectory groups of SA/DP during the 3 years before AMI*** ^**b**^					
Low increasing	957 (68.3)	1	1	1	1
Middle increasing	109 (7.8)	1.86 (1.53–2.27)	1.80 (1.48–2.20)	1.65 (1.35–2.02)	1.60 (1.30–1.96)
Variable	119 (8.5)	1.61 (1.33–1.95)	1.59 (1.31–1.93)	1.44 (1.18–1.75)	1.43 (1.17–1.74)
Constant high	216 (15.4)	1.69 (1.45–1.93)	1.54 (1.32–1.80)	1.49 (1.28–1.74)	1.38 (1.17–1.62)

Abbreviations: AMI = Acute myocardial infarction. SA/DP = Sickness absence/disability pension.

^a^For defining the study population, CMD status prior AMI was based on antidepressant prescription as well as inpatient or specialised outpatient care for depression, anxiety, or stress-related disorders. The outcome measure for CMD (yes/no) after AMI was based on antidepressant prescription.

^b^See section for Statistical methods for details on trajectory variables.

^c^Model 1 = Crude model.

^d^Model 2 = Adjusted for sociodemographic factors.

^e^Model 3 = Adjusted for AMI-related factors and medical factors: comorbidities, including somatic and other mental disorders.

^f^Model 4 = Adjusted for sociodemographic, medical, and AMI-related factors.

In sensitivity analyses, similar findings were observed when we defined the CMD outcome in broader terms, i.e. CMD based on combined information on antidepressant use and health care diagnoses of depression, anxiety, and stress-related disorders (Appendix A in Supplementary Data).

### Post-AMI patterns of work disability and CMD risk

Post-AMI work disability patterns over the course of 1 year were predictive of subsequent CMD risk (Table 4). Relative to the reference group with “constant low” levels of work disability, those with “constant high” levels were associated with the highest rate of CMD, with a fully adjusted HR of 1.76. Individuals who had steeply decreasing levels of work disability—from about 30 days of SA/DP in the first month to no or very low work disability by the third month post-AMI—had a 35% increased CMD risk compared to those with “constant low” work disability levels. The group with “gradually decreasing” levels of post-AMI work disability was associated with a 20% lower rate of CMD compared to the reference group.

**Table 4:** Hazard ratios (HR) and 95% Confidence Intervals (CI) for common mental disorders (CMDs) defined as antidepressant prescription following a diagnosis of AMI from inpatient care in 2008–2010 in Sweden in persons without prior CMD^a^ (N = 10,642).

	CMD	Model 1^c^	Model 2^d^	Model 3^e^	Model 4^f^
	n (%)	HR (95% CI)			
***Trajectory groups of SA/DP during the 12 months after AMI*** ^***b***^					
Constant low	157 (20.5)	1	1	1	1
Steeply decreasing	162 (21.2)	1.43 (1.15–1.78)	1.38 (1.11–1.72)	1.41 (1.13–1.76)	1.35 (1.08–1.69)
Gradually decreasing	211 (27.6)	0.81 (0.66–1.00)	0.79 (0.64–0.98)	0.83 (0.67–1.02)	0.80 (0.65–0.99)
Constant high	235 (30.7)	2.10 (1.71–2.57)	1.92 (1.56–2.36)	1.92 (1.56–2.36)	1.76 (1.43–2.17)

Abbreviations: AMI = Acute myocardial infarction. SA/DP = Sickness absence/disability pension.

^a^For defining the study population, CMD status prior AMI was based on antidepressant prescription as well as inpatient or specialised outpatient care for depression, anxiety, or stress-related disorders. The outcome measure for CMD (yes/no) after AMI was based on antidepressant prescription.

^b^See section for Statistical methods for details on trajectory variables.

^c^Model 1 = Crude model.

^d^Model 2 = Adjusted for sociodemographic factors.

^e^Model 3 = Adjusted for AMI-related factors and medical factors: comorbidities, including somatic and other mental disorders.

fModel 4 = Adjusted for sociodemographic, medical, and AMI-related factors.

Again, sensitivity analyses were performed based on a CMD outcome measure with a broader definition. Regardless of how CMD was defined, the pattern of findings remained stable, with “gradually decreasing” levels associated with a lower risk of CMD while other trajectory groups were associated with greater CMD risk.

## Discussion

### Summary of findings

This is the first study to report that pre- and post-AMI patterns of work disability were associated with subsequent CMDs, even after adjustment for sociodemographic and medical factors. This association could be demonstrated using an exceptionally large, population-based cohort study. Pre-AMI work disability findings indicate that having higher absolute annual levels of SA/DP (>1–12 months) during the 3 years prior AMI were associated with higher risk of subsequent CMD compared to “low increasing” trajectories of work disability. The associations between post-AMI work disability and risk of CMD represent novel findings. Patients with “steeply decreasing” or “constant high” post-AMI work disability trajectories showed higher risk for CMD; conversely, individuals following “gradually decreasing” versus “constant low” levels of work disability were protected against CMD.

The distribution of trajectory groups of pre-AMI work disability are comparable to that published in an earlier paper, wherein the majority of patients had increased annual levels of work disability up until the occurrence of the first AMI^20^. However, no study has to date, investigated work disability trajectories over a 12-month period after AMI and the extent to which they are linked to subsequent CMD risk, which we have shown. High levels of pre- and post-AMI work disability relative to lower levels were robustly associated with a poorer AMI prognosis in terms of CMD risk based on main and sensitivity analyses. To the best of our knowledge this is the first time such an association is shown. One explanation for these findings is that the measure of SA/DP captures work incapacity due to a number of different diseases, which in turn are associated with higher susceptibility to CMD^27,28^. However, the aforementioned associations remained even after adjustment for potential confounding, including numerous somatic as well as psychiatric comorbidities, suggesting that having higher versus lower levels of pre-AMI work disability *per se* may have a modest but negative influence on CMD outcomes. Still, we acknowledge the possibility of residual confounding as we were unable to account for undetected disorders as well as diagnoses made in primary care. Nonetheless, the mechanism underlying the associations may be tied to social isolation or lack of social engagement, which are associated with depressive symptoms^15,23,29^. Moreover, poorer health behaviour, i.e. less physical exercise, smoking, and alcohol abuse, which is influenced by social isolation and has been linked to sickness absence, may play a role^29,30^. Post-AMI associations—where higher levels of work disability resulted in higher rates of CMD—could be interpreted in a similar fashion. High levels of work disability after AMI may also be a marker of poor vocational rehabilitation or difficulties finding or continuing work when one wants to return to work after an AMI. Besides absolute levels of post-AMI work disability, the shape of trajectories was particularly important as a CMD determinant, which we discuss below.

Of note, with regard to the association between post-AMI work disability trajectories and CMD, “gradually decreasing” trajectories were associated with the lowest rate of CMD, even more so than those with “constant low” and “steeply decreasing” levels of work disability. “Constant low” and “steeply decreasing” levels of work disability may be indicative of inadequate rehabilitation time in terms of time off work after an AMI, whereas gradually decreasing levels may reflect the necessary time for recovery. Further scrutiny revealed that the association attenuated more so upon controlling for sociodemographic factors, indicating that some patients may have returned to work earlier than necessary, due perhaps to financial strain. This in turn may have led to an increased risk of subsequent CMD. Importantly, our findings support the current recommendation of 4–8 weeks of sick leave after an AMI without complications in Sweden^4^, though tapering off sick leave until month 9 to 11 after an AMI may be optimal for keeping CMD at bay.

Altogether, our study highlights the heterogeneity of longitudinal patterns of work disability before and after AMI, and sheds light on the relationship between these patterns with subsequent CMD risk, which have so far been unexamined in the literature.

### Strengths and limitations

Strengths of the study include the prospective design, thereby eliminating risk of recall bias. The register-based nature of the study allowed for linkage of data between several national registers, which allowed adjustment of potential confounding factors such as relevant sociodemographic factors and medical conditions. The use of population-based longitudinal data of the exposure and outcome was also advantageous. Moreover, with such data from the Prescribed Drug Register and the National Patient Register, we were able to restrict the sample to individuals without prior CMDs, thus minimizing, though not wholly, the risk of reverse causation for the association between work disability patterns and subsequent CMD risk. We were also able to perform sensitivity analyses wherein CMD was more broadly defined, which allowed for a deeper inspection of the influence of work disability as a way to reduce bias due to outcome misclassification. However, while registers are rich with information, we could only construct measures insofar as what the data allowed.

A limitation of the study was that our CMD measures in main and sensitivity analyses did not take into account primary care data, which was unavailable. Thus, there is a risk of misclassification of CMD, which may bias the association towards the null. Another limitation of the study had to do with the data on work disability; information on the first 14 days of sickness absence for employed persons is unavailable, and this may have led to an underestimation of the length of SA/DP. However, as we have included the patients with AMI as our study population, and 4–8 weeks of SA is recommended for AMI without complications in Sweden, therefore, the majority of them is likely to have had longer SA/DP during the post-AMI period. Moreover, this lack of information on short term work disability, i.e. the first two weeks, is only true for employed on SA only. As we included both SA and DP in our analyses and mean work disability levels are ranging between much higher levels, i.e. 1 and 12 months per year, it is unlikely that this lack of information has led to any considerable alterations in the measurements. Moreover, as this lack applies to all trajectory groups, the main patterns have not been affected. Some unmeasured residual confounding might be present due to e.g. the lack of data on measures related to vocational rehabilitation or negative job exposures after an AMI. Still, introduction of such potential confounders would not have altered the estimates considerably as the analyses are already controlled for factors strongly associated with these markers, namely educational level and area of living. Finally, the examination of post-AMI work disability trajectories was based on a smaller population of patients who had survived for at least 1 year after the AMI; however, characteristics were very similar between the main and “surviving” cohorts, indicating that bias arising from a healthy cohort effect was unlikely.

Considering the quality of the data and the design of the study, we would consider our findings to be widely generalizable, however, depending upon differences in the social insurance system, which provides compensation for work disability, and variability in the healthcare system, one should interpret the results with caution. Further research considering such differences be advisable to replicate our findings.

## Conclusions

This is the first study to demonstrate that pre- and post-AMI patterns of work disability are associated with risk of subsequent CMD in AMI patients in the following 5 to 6 years. In particular, both increasing and high persistent levels of pre-AMI work disability are associated with higher risk of CMD, while post-AMI findings suggest that patients who gradually decreased their work disability had a favourable CMD prognosis. This study highlights the importance of capturing the longitudinal course of work disability before and after an AMI, and that these patterns of work disability as predictors of AMI prognosis in terms of CMD risk should be considered in clinical practice.

## Supplementary information

Common mental disorders (CMDs) following a diagnosis of acute myocardial infarction from inpatient care in 2008-2010 in Sweden in those without previous CMD
