# Supplementary material for: Work disability patterns before and after incident acute myocardial infarction and subsequent risk of common mental disorders: A Swedish cohort study
Source: Sci Rep. 2019 Nov 6;9:16086. doi: 10.1038/s41598-019-52487-w (PMC6834568; doi:10.1038/s41598-019-52487-w)
Supplement: Supplementary file 1 — Common mental disorders (CMDs) following a diagnosis of acute myocardial infarction from inpatient care in 2008-2010 in Sweden in those without previous CMD [file 41598_2019_52487_MOESM1_ESM.docx]

# Title page

# Work disability patterns before and after incident acute myocardial infarction and subsequent risk of common mental disorders: A Swedish cohort study

**Authors:**

**Bokenberger K., PhD**^1^ **- Postdoctoral Researcher**

**Rahman S., PhD**^1^ **- Postdoctoral Researcher**

**Wang M., PhD**^1^ **- Postdoctoral Researcher**

**Vaez M., PhD**^1^ **- Associate Professor**

**Dorner T.E., MD, PhD**^1,2^ **- Associate Professor**

Helgesson **M., PhD**^1^ **- Postdoctoral Researcher**

**Ivert T., MD, PhD**^3^ **- Senior Researcher**

**Mittendorfer-Rutz E., PhD**^1^ **- Senior Researcher**

^1^ Division of Insurance Medicine, Department of Clinical Neuroscience, Karolinska Institutet, Stockholm, Sweden

^2^ Department of Social and Preventive Medicine, Centre for Public Health, Medical University of Vienna, Vienna, Austria

^3^ Department of Molecular Medicine and Surgery, Karolinska Institutet and Heart and Vascular Theme, Karolinska University Hospital, Stockholm, Sweden

**Funding:** Swedish Research Council (2015-02292).

**Corresponding author**:

Dr. Syed Rahman

Division of Insurance Medicine, Department of Clinical Neuroscience,

Karolinska Institutet, SE-171 77 Stockholm, Sweden

E-mail: Syed.rahman@ki.se; Tel.: +46-8-524 832 24; Fax: +46-8- 524 832 05

**Supplementary file for online publication**

| **Appendix A.** Hazard ratios (HR) and 95% Confidence Intervals (CI) for common mental disorders (CMDs) defined as antidepressant prescription and health care diagnosis of depression, anxiety, and stress-related disorders following a diagnosis of acute myocardial infarction (AMI) from inpatient care in 2008-2010 in Sweden in those without previous CMD^a^ | | | | | |
| --- | --- | --- | --- | --- | --- |
|  | **CMD** | Model 1^e^ | Model 2^f^ | Model 3^g^ | Model 4^h^ |
|  | n (%) | HR (95% CI) | | | |
| ***Trajectory groups of SA/DP during the 3 years before AMI*^b,c^** | | | | | |
| Low increasing | 1,054 (68.4) | 1 | 1 | 1 | 1 |
| Middle increasing | 113 (7.3) | 1.74  (1.43-2.12) | 1.70  (1.40-2.06) | 1.56  (1.28-1.91) | 1.52  (1.24-1.85) |
| Variable | 130 (8.4) | 1.61  (1.34-1.93) | 1.61  (1.33-1.93) | 1.44  (1.20-1.74) | 1.45  (1.20-1.75) |
| Constant high | 243 (15.8) | 1.73  (1.51-1.99) | 1.61  (1.39-1.86) | 1.55  (1.34-1.79) | 1.45  (1.24-1.69) |
| ***Trajectory groups of SA/DP during the 12 months after AMI*^b,d^** | | | | | |
| Constant low | 171 (20.4) | 1 | 1 | 1 | 1 |
| Steeply decreasing | 174 (20.7) | 1.40  (1.14-1.73) | 1.36  (1.10-1.68) | 1.39  (1.12-1.72) | 1.34  (1.08-1.66) |
| Gradually decreasing | 235 (28.0) | 0.82  (0.68-1.00) | 0.81  (0.66-0.99) | 0.84  (0.69-1.02) | 0.81  (0.67-1.00) |
| Constant high | 259 (30.9) | 2.11  (1.74-2.56) | 1.94  (1.59-2.36) | 1.94  (1.59-2.36) | 1.78  (1.46-2.18) |

Abbreviations: AMI=Acute myocardial infarction. SA/DP=Sickness absence/disability pension.

a. For defining the study population, CMD status prior AMI was based on antidepressant prescription as well as inpatient or specialised outpatient care for depression, anxiety, or stress-related disorders. The outcome measure for CMD (yes/no) after AMI was based on antidepressant prescription.

b. See section for Statistical methods for details on trajectory variables.

c. Based on main cohort of 11,493 AMI patients without previous CMD.

d. Based on sub-cohort of 10,642 AMI patients still alive and who had not developed CMD within 1 year after AMI.

e. Model 1=Crude model.

f. Model 2=Adjusted for sociodemographic factors.

g. Model 3=Adjusted for AMI-related factors and medical factors: comorbidities, including somatic and other mental disorders.

h. Model 4=Adjusted for sociodemographic, medical, and AMI-related factors.
